# Serum LAPTM4B as a Potential Diagnostic and Prognostic Biomarker for Breast Cancer

**DOI:** 10.1155/2022/6786351

**Published:** 2022-11-30

**Authors:** Lu Wang, Yating Wang, Qingyun Zhang

**Affiliations:** Department of Clinical Laboratory, Key Laboratory of Carcinogenesis and Translational Research, Ministry of Education, Peking University Cancer Hospital & Institute, Beijing 100142, China

## Abstract

**Background:**

Lysosome-associated protein transmembrane-4 beta (LAPTM4B) is an integral membrane protein overexpressed in various cancers and may function as a prognostic tumor marker. The present study is aimed at understanding the clinical significance of serum LAPTM4B in breast cancer (BC).

**Methods:**

Serum LAPTM4B level was evaluated in 426 BC patients, 40 benign breast disease, and 80 healthy controls by ELISA. We used the receiver operator characteristic (ROC) curve to assess the diagnostic significance. 46 BC patients were recruited to monitor the dynamic change of serum LAPTM4B during adjuvant therapy (AT). In addition, sera from a subset of 330 patients undergoing AT, including anti-HER2 treatment, were collected to evaluate the association between LAPTM4B levels and AT efficacy. Descriptive and explorative statistical analyses were used to assess LAPTM4 B's potential as a diagnostic and prognostic marker in BC.

**Results:**

Serum LAPTM4B level was significantly increased in BC patients than benign group and controls. It could well discriminate BC from healthy controls with diagnostic accuracy with an AUC of 0.912, a sensitivity of 85.9%, and a specificity of 83.8%. Compared with pre-AT, serum LAPTM4B concentration remarkably decreased after AT. In addition, patients in the invalid response group (PD + SD) showed higher LAPTM4B levels than the valid response group (PR + CR).

**Conclusion:**

Our results proposed that serum LAPTM4B had a high diagnostic and prognostic impact as a circulating biomarker in BC.

## 1. Introduction

Breast cancer is the most frequently diagnosed cancer and ranks the second cause of cancer-related death in Chinese women [1]. This disease is characterized by enormous heterogeneity and classified concerning the presence or absence of these receptors as luminal A (estrogen receptor, ER, and/or progesterone receptor, PR positive, and human epidermal growth receptor 2, HER2 negative), luminal B (ER and/or PR positive and HER2 positive), HER2-enriched (ER and PR negative and HER2 positive), and basal-like (triple negative breast cancer-ER, PR, and HER2 negative) [2]. The past and ongoing research have been concerned with identifying biomarkers for diagnosis, especially for early detection and therapy selection of BC. Serological biomarkers as noninvasive protocols have advantages in detection convenience over other types. Currently, the combination of one MUC-1 family protein (such as CA15-3, BR27.29, MCA, and CA549) and carcinoembryonic antigen (CEA) is the recommended serum marker panel in BC patients [3]. However, the lack of sensitivity, especially for early-stage disease [4], and appearance of spurious rises after treatment [5–8] and may preclude the clinical use of these makers.

The oncogene lysosome-associated protein transmembrane-4*β* (LAPTM4B) was first cloned in hepatocellular carcinoma (HCC) [9], and the polymorphism region in the 5′-UTR of the gene is associated with tumor susceptibility [10–19]. LAPTM4B gene is mapped to chromosome 8q22.1 and encodes an integral membrane protein with four transmembrane regions [20] and is upregulated in various solid tumor tissues associated with poor prognosis, including breast cancer, NSCLC, ovarian cancer, HCC, gastric cancer, and PDAC [21–27]. Based on our mechanism study of LAPTM4B, transcription factors and microRNA could bind to LAPTM4B promoter regions to regulate its expression and exert oncogenic effects in breast cancer [28–30]. LAPTM4B contributes to tumorigenesis by promoting cell proliferation, boosting invasion, resisting apoptosis, initiating autophagy, and assisting drug resistance [31]. Moreover, LAPTM4B gene copy number gain is associated with a poor prognosis for anthracycline-based chemotherapy in hormone receptor-negative BC [32]. However, the significance of circulating LAPTM4B levels in BC and its relationship with prognosis remain unclear.

Here, we examine the serum levels of LAPTM4B in BC and control for diagnostic accuracy. We explore the prognostic potential of serum LAPTM4B as a monitoring tool for response in BC patients to adjuvant treatment.

## 2. Material and Methods

### 2.1. Ethics

The study was conducted in compliance with the guidelines of the Helsinki Declaration of 2013 and approved by the Beijing Cancer Hospital Ethics Committee. Informed consent was obtained from all individual participants of the present study.

### 2.2. Patients and Study Design

**Figure 1:**
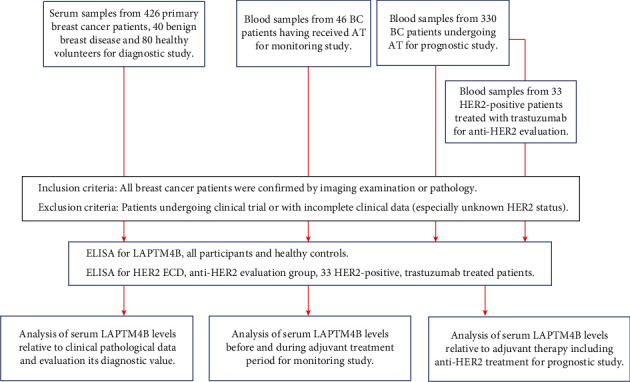
Flow chart of patients and healthy controls recruited to the study. For the diagnostic serum study, blood samples were collected from primary breast cancer patients (*n* = 426), benign breast disease (*n* = 40), and healthy controls (*n* = 80) attending Beijing Cancer Hospital. For the monitoring study, patients who had received adjuvant therapy were enrolled, and their blood samples were collected before and after treatment (*n* = 46). In addition, patients undergoing their adjuvant cycles in Beijing Cancer hospital were selected for efficacy evaluation (*n* = 330), containing 33 HER2-positive patients receiving trastuzumab therapy.

For the diagnostic serum study, 426 breast cancer patients were recruited between April 2020 and September 2020 at Beijing Cancer Hospital. We also collected 40 benign breast disease samples including benign breast tumors and breast adenosis. Clinical parameter data of BC, benign breast disease, and healthy controls are present in Table 1. For the monitoring study, 46 BC patients who received adjuvant therapy were enrolled in the AT group, and their blood samples were collected before and after treatment, respectively. For the prognostic study, 330 BC patients undergoing adjuvant cycles in Beijing Cancer hospital were selected. Among them, 33 HER2-positive BC patients received Herceptin (trastuzumab) therapy. The disease stages were determined according to the American Joint Committee on Cancer (AJCC) TNM (tumor-node-metastasis) classification [33]. The treatment effect was assessed based on Response Evaluation Criteria in Solid Tumors (RECIST) guidelines [34]. The flow chart of the study design is presented in Figure 1.

### 2.3. Clinical Assay for Serum LAPTM4B and HER2-ECD

A total of 5 mL peripheral venous blood was obtained and then centrifuged at 1300 g for 10 min. The supernatant was centrifuged at 10,000 g for 10 min to eradicate cellular contaminants. The serum was aliquoted and snap-frozen at -80°C until use.

Serum LAPTM4B level was measured by quantitative human LAPTM4B sandwich enzyme-linked immunosorbent kits (ELISA) (LifeSpan BioSciences, Inc., Seattle, USA) following the manufacturer's instructions. The serum HER2-ECD levels from HER2-positive patients were detected using a commercial ELISA kit (Shanghai Fengshou Industrial Co., Ltd., Shanghai, China) according to the manufacturer's instructions. In brief, a microtiter plate coated with capture antibody was incubated with 100 *μ*l serum for 1 h at 37°C. After washing, the detection antibody was added and incubated for 1 h at room temperature. Adequate washing was carried out after each step. Following avidin-horseradish peroxidase-conjugated secondary antibody and TMB substrate solution, a stop solution was added to terminate the reaction. Finally, the absorbance was determined at 450 nm in a microplate reader (Bio-Rad, Hercules, CA). The professional software ELISACalc capable of generating a four-parameter logistic (4-PL) curve fit was used to calculate the serum makers' concentrations.

### 2.4. Statistical Analysis

Statistical analyses were performed using the Statistical Software Package for the Social Sciences (SPSS software version19.0, SPSS) and GraphPad Prism 7.0. Values of *p* < 0.05 were considered statistically significant. Concentrations of serum markers were described using the median and interquartile range (IQR) when the data did not meet the normal distribution. Mann–Whitney *U* tests were used for the comparison of two independent groups. Comparisons of paired samples were performed by applying Wilcoxon singed-rank test. One-way ANOVA was used to measure the LAPTM4B levels between BC, benign breast disease, and healthy controls. The receiver operating characteristic (ROC) curve was plotted, and the area under the curve (AUC) with its corresponding 95% confidence interval (CI) was calculated as an accuracy index for evaluating the diagnostic performance.

## 3. Results

### 3.1. Analysis of Serum LAPTM4B Levels in Subjects

**Figure 2:**
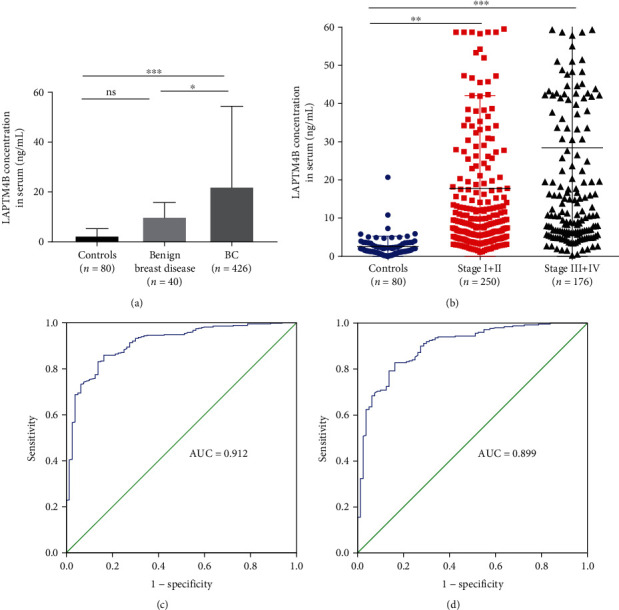
Diagnostic potential of serum LAPTM4B in BC. (a) The concentration of LAPTM4B in the serum of BC, benign breast disease, and healthy controls. Each bar represents the median values ± quartile values. (b) The concentration of LAPTM4B in the serum of stage I + II and stage III + IV BC patients and healthy controls. Receiver operating characteristic (ROC) curve analysis for the diagnostic value of LAPTM4B in all BC patients (c) and early stage (stages I-II) (d). ns: no significance. ^∗∗^*p* < 0.05, ^∗∗^*p* < 0.01, and ^∗∗∗^*p* < 0.001.

Firstly, we analyzed the serum LAPTMB levels of a large set of 426 primary breast cancer patients, 40 benign breast disease, and 80 healthy control subjects. There was no difference in age and gender among the groups. The LAPTM4B level in the BC group was significantly higher than benign breast disease and healthy controls (Table 1, Figure 2(a)). As shown in Table 2 and Figure 2(b), the median level of LAPTM4B in the healthy control group was significantly lower than in the stage I + II (*p* < 0.01) and III + IV BC patients (*p* < 0.001). In addition, we sought to characterize LAPTM4B levels in 426 BC samples in the context of various clinicopathological variables (Table 3). As a result, no association was observed between LAPTM4B and menopausal status, hypertension, diabetes, tumor size, and HER2 status. However, there were significant associations between LAPTM4B levels and classic variables including age (*p* = 0.020), histological type (invasive ductal carcinoma, IDC) (*p* = 0.021), TNM stage (*p* = 0.001), nodal metastasis (*p* < 0.001), distant metastasis (*p* = 0.001), and Ki-67 status (*p* = 0.034). In addition, patients with ER and PR negative status exhibited higher LAPTM4B levels than others. As expected, the serum LAPTM4B level of triple-negative BC (TNBC) patients was significantly increased than other subtypes (*p* = 0.006).

### 3.2. Diagnostic Value of LAPTM4B for Primary Breast Cancer

To better assess the diagnostic value of serum LAPTM4B in BC, ROC curve was plotted, and AUC was calculated. Serum LAPTM4B showed potential as a discriminator between BC and healthy controls with excellent accuracy (AUC = 0.912, 95% CI 0.880-0.945, *p* < 0.001) (Figure 2(c)). At the cut-off point of 3.67 ng/mL, the positive rate was 88.73%, and the optimal sensitivity and specificity were 0.859 and 0.838, respectively. In addition, subgroup analysis of early stage of BC (stage I + II) confirms good AUC values versus healthy controls (AUC = 0.899, 95% CI 0.861-0.938, *p* < 0.001) (Figure 2(d)), indicating its performance for early diagnosis of BC.

### 3.3. Clinical Values of Changes of Serum LAPTM4B Levels in Adjuvant Therapy

#### 3.3.1. Monitoring of LAPTM4B during the Course of Adjuvant Treatment

**Figure 3:**
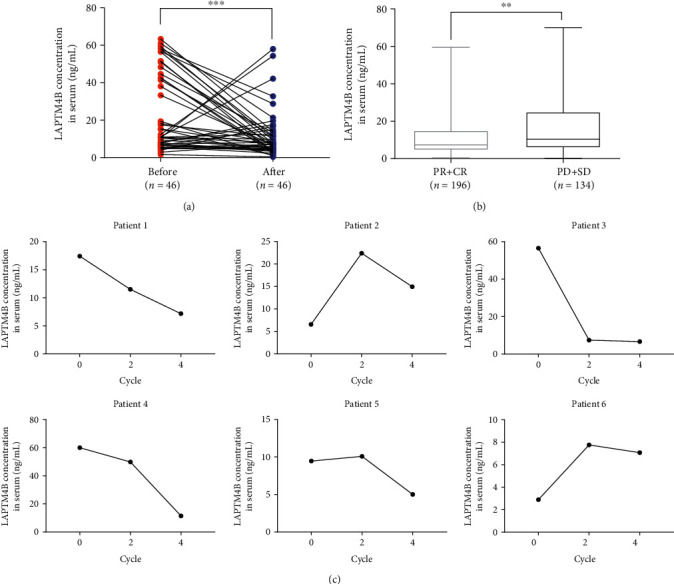
Changes of serum LAPTMB levels in BC patients with adjuvant therapy. (a) Comparison of serum LAPTM4B levels pre- and post-AT. (b) The concentrations of LAPTM4B of different AT efficacy groups. (c) The dynamic changes of serum LAPTM4B levels in 6 typical patients. ^∗∗^*p* < 0.01 and ^∗∗∗^*p* < 0.001.

46 BC patients were screened for changes in serum LAPTM4B levels before and after two cycles of adjuvant therapy (including chemotherapy, endocrine, and anti-HER2 treatment). Matched pre-AT and post-AT serum levels of LAPTM4B were measured, and the results showed that LAPTM4B levels sharply decreased after AT (*p* = 0.001, Table 4, Figure 3(a)).

#### 3.3.2. Prognostic Impact of Serum LAPTM4B

To evaluate the LAPTM4B levels and efficacy of adjuvant therapy, we investigated 330 BC patients undergoing their adjuvant therapy cycles in Beijing Cancer Hospital, including 191 endocrine therapy, 106 chemotherapy or radiotherapy, and 33 anti-HER2 therapy. They were categorized into progressive-disease (PD) plus stable-disease (SD) group and partial-response (PR) plus complete-response (CR) based on the results of CT scans obtained every two cycles of AT. The results revealed that the serum LAPTM4B level of the PD + SD group was significantly higher than that of the PR + CR group (*p* = 0.004, Table 5, Figure 3(b)). The differences in LAPTM4B levels between PD + SD and PR + CR group in different treatment methods are shown in Supplementary Table S1. Moreover, as seen in Table 3, BC patients with clinically confirmed disease recurrence exhibited much higher LAPTM4B levels than the others (*p* < 0.001).

#### 3.3.3. Dynamic Changes of Serum LAPTM4B Levels in 6 Typical Cases

In notice, among the 46 patients, six patients were screened for obtaining their blood samples before and after two and four cycles of AT for constant monitoring. All subjects received four cycles of AT during the course of the study. As shown in Figure 3(c), the initial serum LAPTM4B of patient 3 receiving trastuzumab combined with pertuzumab decreased immediately after two cycles of AT and was maintained until the end of four treatment cycles. The efficacy of AT was evaluated as CR. After two cycles of AT, the serum LAPTM4B levels of patients 1 and 4 receiving albumin combined with paclitaxel chemotherapy were lower than before AT, which was still relatively high. It continued to decrease significantly till the end of the fourth cycle. The efficacy evaluation of these two patients was both PR, whereas after four treatment cycles, the serum LAPTM4B levels of patients 2 and 6 who had received albumin combined with paclitaxel chemotherapy underwent an elevation higher than pre-AT, indicating poor treatment efficacy. At this time, imaging examination also showed tumor metastasis, and the efficacy was evaluated as PD. The serum LAPTM4B level of post-AT was lower than before in patient 5 receiving trastuzumab combined with pertuzumab. Although it increased slightly after the second AT cycle, the overall change was relatively stable. The efficacy of AT was also evaluated as SD.

Therefore, the serum LAPTM4B level of the six patients has a good consistency with its treatment effect, but no effect of treatment on its LAPTM4B level has been found, and further large-sample data support is needed.

### 3.4. Prognostic Value of Serum LAPTM4B in Anti-HER2 Therapy

Trastuzumab (Herceptin) is a biologically active, humanized monoclonal antibody which has been reported to improve the survival rates for HER2/neu-positive BC [35]. To investigate the clinical significance of LAPTM4B in HER2-targeted sensitivity, we selected a cohort of 33 HER2-positive BC patients who had received trastuzumab either as front-line or salvage treatment. As shown in Table 6, 18 patients were sensitive to Herceptin, and 15 were resistant to Herceptin. As expected, serum HER2-ECD levels in Herceptin-resistant patients were significantly higher than in sensitive patients (*p* = 0.021). Moreover, the level of serum LAPTM4B was consistent with the changing trend of HER2-ECD. Compared with sensitive patients, a higher proportion of patients who were resistant to anti-HER2 treatment exhibited high LAPTM4B levels (*p* = 0.027).

Therefore, these results indicated that serum LAPTM4B might be a valuable biomarker for tracking disease and monitoring adjuvant treatment responses, including anti-HER2 therapy.

## 4. Discussion

For decades, the survival rates of BC have been significantly improved due to the development of treatment strategies. The optimal BC treatment is surgery accompanied by adjuvant therapy, referring to chemotherapy, radiotherapy, hormone, and HER2-targeted therapy. However, poor prognosis of TNBC and drug resistance presents major obstacles for BC management. As reported, 30% of early BC still advance to metastatic breast cancer (MBC) [36], while the median survival of MBC is generally between 24 and 30 months after metastasis [37, 38]. Therefore, it is critical to establish a diagnostic method for the early detection of BC. Several studies reported that the overexpression of LAPTM4B-35 in BC tissues might contribute to tumor progression and poor prognosis [21, 39, 40].

Furthermore, we have revealed that transcription factor AP-4 and microRNA-132-3p could bind to LAPTM4B protomer regions to regulate breast cancer cell proliferation and metastasis and assist drug assistance in vitro and vivo through c-myc, EMT, and PI3K-AKT signal pathways [29, 30]. Thus, it represents an attractive therapeutic target for BC. Based on the previous study, LAPTM4B protein, which belongs to the mammalian-4-tetratransmembrane spanning protein superfamily, may be released into blood from tumor cells in the form of exosomes and was highly increased in serum of HCC [41]. Moreover, our results have also found that the serum level of LAPTM4B was significantly associated with the tumor progression and treatment effects of lung adenocarcinoma [42]. However, the clinical value of circulating LAPTM4B for BC remained undefined.

For the present study, we screened the serum level of LAPTM4B in BC and age-matched healthy controls using ELISA. The results showed that LAPTM4B was markedly increased in sera from BC patients compared with benign breast disease and normal controls. However, we did not find a significant difference between benign and healthy controls. It may be caused by the small size of benign sample, and larger collaborative studies needed to validate the results. For the ROC analysis, circulating LAPTM4B could differentiate between BC patients and early BC (AJCC stages I-II) from healthy controls with excellent AUCs suggesting its diagnostic value as a noninvasive serum marker. Serum LAPTM4B reached a diagnostic accuracy with an optimal sensitivity of 85.9% and specificity of 83.8% in our collective. As reported, the sensitivity of CEA in the diagnosis of MBC was 46-53%, the sensitivity of CA15-3 was 54%-87%, and combining both of them was up to 64%-94% [43]. It seems that the LAPTM4B assay is more sensitive and indicative of the change of tumor burden than the single commonly used diagnostic biomarker for BC. In addition, LAPTM4B level in sera was associated with age, histological type (infiltrating ductal, IDC), TNM stage, nodal metastasis, distant metastasis, Ki-67 status, and recurrence, which was consistent with the role for LAPTM4B in BC tissues [21, 39]. In addition, TNBC is characterized by the absence or low expression of ER, PR, and HER2. Patients with TNBC often exhibit unfavorable histopathologic features at diagnosis and are associated with a shorter median time to relapse and death [44, 45]. Interestingly, serum LAPTM4B concentration was significantly increased in TNBC than in other subtypes, which may provide novel molecular targets for therapy selection.

The study further explores the potential impacts of general BC treatment involving chemotherapy, endocrine, and targeted molecular therapy on serum LAPTM4B. Similar to our previous findings of declined serum LAPTM4B levels in postchemotherapy samples in lung adenocarcinoma [42], serum LAPTM4B levels were significantly decreased in BC patients after adjuvant therapy. The decreased trends support that serum LAPTM4B levels have considerable correlations with tumor dynamics. Moreover, in this study, monitoring serum levels during the adjuvant treatment period identified serum LAPTM4B levels correlated with efficacy evaluation of AT. Patients who did not benefit from AT were likelier to have elevated LAPTM4B levels. Li et al. have reported that LAPTM4B could act on anthracycline trafficking by reducing drug entry to nucleus and decreasing drug-induced DNA damage, which leads to resistance and recurrence of BC [46]. Rusz et al. also found that LAPTM4B gene copy number gain was associated with an inferior response to anthracycline-based chemotherapy in hormone receptor-negative BC [32]. These results confirm the possible role of LAPTM4B gene in anthracycline resistance.

Moreover, it is interesting to note that chromosome 8 polysomy, where the LAPTM4B gene is located, was present in 39% HER2-positive tumors and 30.2% HER2-negative tumors [47]. The high LAPTM4B expression contributed to the resistance to neoadjuvant chemotherapy in HER2-negative BC [48]. Based on these findings, we wanted to explore further the potential role of LAPTM4B in HER2-positive BC. The extracellular domain (ECD) of HER2 is released into blood by a proteolytic cleavage, known as “shedding” [49]. It has been revealed that HER2-ECD could indicate cancer progression and therapy response, particularly anti-HER2 therapy [50]. Trastuzumab, a humanized monoclonal antibody binding to HER2-ECD, has been reported to improve the survival rates for HER2-positive BC patients [35]. Consistent with the previous study, our data demonstrated that serum HER2-ECD levels significantly declined in HER2-positive patients who benefited from trastuzumab treatment, accompanied by a significant decrease of serum LAPTM4B levels, from which we could conclude that serum LAPTM4B also has potential as a new surveillance tool for patients with HER2 positive to monitor ongoing response to trastuzumab therapy. The prevalent mechanism leading to trastuzumab resistance is the activation of PI3K/AKT pathway [51], which overlaps with regulatory signals of LAPTM4B gene. Thus, we could make efforts to increase the sensitivity of anti-HER2 treatment by targeting LAPTM4B.

The retrospective study is somewhat limited due to the relatively low number of patients, which reflects the challenges in collecting serial serum samples from different strategies, including chemotherapy, endocrine, and targeted molecular therapy. In addition, further mechanism research should elucidate the roles of LAPTM4B on the efficacy of anti-HER2 treatment.

## 5. Conclusions

Taken together, this study extends the findings about the serum levels of LAPTM4B in breast cancer patients. Our data provide complementary information on its diagnostic value. From another respect, the serum level of LAPTM4B is significantly associated with tumor progression and efficacy evaluation, suggesting its roles in monitoring treatment and assessing tumor dynamics.

## Figures and Tables

**Table 1:** Characteristics of study participants.

	Healthy control (*n* = 80)	Benign breast disease (*n* = 40)	Breast cancer (*n* = 426)	*p* value
Age (median, range)	48 (23-61)	45 (23-81)	55 (25-85)	0.14
Gender (F/M)	80/0	40/0	426/0	—
LAPTM4B (ng/mL, median, IQR)	2.01 (2.36)	8.93 (7.59)	10.01 (23.22)	<0.001

**Table 2:** Comparison of serum LAPTM4B levels in breast cancer patients and normal controls (median (IQR)).

Group	Number	LAPTM4B (ng/mL)	*p* value
Controls	80	2.01 (2.36)	*p* < 0.001
BC (stage I + II)	250	8.85 (15.40)	
BC (stage III + IV)	176	13.91 (35.33)	

**Table 3:** Correlation of serum LAPTM4B with clinical indicators in breast cancer patients (median (IQR)).

Variables	Number	LAPTM4B (ng/mL)	*p* value
*Age*			
≤ 55	226	9.31 (14.68)	0.020
> 55	200	11.14 (31.12)	
*Menopausal status*			
Premenopausal	211	9.76 (15.39)	0.206
Postmenopausal	215	10.53 (28.36)	
*Hypertension*			
-ve	116	9.78 (19.83)	0.302
+ve	310	12.55 (30.15)	
*Diabetes*			
-ve	386	10.73 (24.75)	0.192
+ve	40	7.21 (15.92)	
*Histological type*			
IDC	328	10.77 (27.63)	0.021
Others	98	8.25 (14.75)	
*TNM stage*			
I + II	250	8.85 (15.40)	0.001
III + IV	176	13.91 (35.33)	
*Tumor size*			
≥ 20 mm	208	10.59 (28.08)	0.694
< 20 mm	218	9.78 (20.91)	
*Nodal status*			
-ve	237	8.49 (15.46)	<0.001
+ve	189	13.84 (35.57)	
*Distant metastasis status*			
-ve	277	9.00 (18.25)	0.001
+ve	149	15.16 (34.98)	
*Recurrence*			
-ve	301	8.59 (14.77)	<0.001
+ve	125	16.70 (34.78)	
*ER*			
-ve	97	15.97 (38.98)	<0.001
+ve	329	9.00 (16.03)	
*PR*			
-ve	134	13.91 (35.59)	0.003
+ve	292	8.99 (18.18)	
*HER2*			
-ve	115	11.38 (21.58)	0.996
+ve	311	9.76 (24.79)	
*Ki-67*			
-ve	52	7.12 (11.09)	0.034
+ve	374	10.66 (27.55)	
*Triple-negative*			
Yes	19	25.68 (60.32)	0.006
No	407	9.51 (21.83)	

**Table 4:** Changes of serum LAPTM4B levels in BC patients before and after two cycles of adjuvant therapy (median (IQR)).

Group	Number	LAPTM4B (ng/mL)		*p* value
		Before AT	After AT	
Adjuvant therapy patients	46	11.06 (36.48)	7.25 (9.32)	0.001

**Table 5:** Comparison of serum LAPTM4B levels in BC patients between adjuvant therapy efficacy groups (median (IQR)).

Efficacy	Number	LAPTM4B (ng/mL)	*p* value
PR + CR	196	7.39 (10.60)	0.004
PD + SD	134	10.39 (19.25)	

**Table 6:** High serum LAPTM4B concentration is inversely associated with anti-HER2 sensitivity in HER2-positive breast cancer (median (IQR)).

Anti-HER2	Number	HER2-ECD (ng/mL)	*p* value	LAPTM4B (ng/mL)	*p* value
Responder	18	7.39(10.60)	0.021	6.57(5.61)	0.027
Nonresponder	15	10.39(19.25)		9.99(19.69)	

## Data Availability

Data is available on request from the authors.

## Abbreviations

AT: Adjuvant therapy
AUC: Area under the curve
BC: Breast cancer
CEA: Carcinoembryonic antigen
CI: Confidence interval
ECD: The extracellular domain
ELISA: Enzyme-linked immunosorbent assay
ER: Estrogen receptor
HCC: Hepatocellular carcinoma
HER2: Human epidermal growth receptor 2
LAPTM4B: Lysosome-associated protein transmembrane-4 beta
MBC: Metastatic breast cancer
NSCLC: Non-small-cell lung cancer
PDAC: Pancreatic ductal adenocarcinoma
PR: Progesterone receptor
ROC: Receiver operating characteristic
TNBC: Triple-negative breast cancer.

## Supplementary Materials

Supplementary Materials Supplementary Table S1: comparison of serum LAPTM4B levels in BC patients between adjuvant therapy efficacy groups (median (IQR)).
