# Supplementary material for: Serum LAPTM4B as a Potential Diagnostic and Prognostic Biomarker for Breast Cancer
Source: Biomed Res Int. 2022 Nov 30;2022:6786351. doi: 10.1155/2022/6786351 (PMC9729050; doi:10.1155/2022/6786351)
Supplement: Supplementary Materials — Supplementary Table S1: comparison of serum LAPTM4B levels in BC patients between adjuvant therapy efficacy groups (median (IQR)). [file 6786351.f1.docx]

**Table S1. Comparison of serum LAPTM4B levels in BC patients between adjuvant therapy efficacy groups (median (IQR)).**

| **Groups** | **Endocrine therapy** | | | **Chemotherapy** | | | **Anti-HER2 therapy** | | |
| --- | --- | --- | --- | --- | --- | --- | --- | --- | --- |
| **Efficacy** | **Number** | **LAPTM4B**  **(ng/mL)** | ***P* value** | **Number** | **LAPTM4B**  **(ng/mL)** | ***P* value** | **Number** | **LAPTM4B**  **(ng/mL)** | ***P* value** |
| PR+CR | 126 | 7.26(12.33) | 0.005 | 52 | 7.66(10.39) | 0.277 | 18 | 6.57(5.61) | 0.027 |
| PD+SD | 65 | 13.22(22.18) |  | 54 | 10.15(28.92) |  | 15 | 9.99(19.69) |  |
